# The association between lifelines diet score (LLDS) with depression and quality of life in Iranian adolescent girls

**DOI:** 10.1186/s12937-024-00913-9

**Published:** 2024-02-15

**Authors:** Zahra Darabi, Abbas Ali Sangouni, Majid Ghayour-Mobarhan, Gordon A. Ferns, Sayyed Saeid Khayyatzadeh

**Affiliations:** 1 Research Center for Food Hygiene and Safety, School of Public Health, Shahid Sadoughi University of Medical Sciences, Yazd, Iran; 2 Department of Nutrition, School of Public Health, Shahid Sadoughi University of Medical Sciences, Yazd, Iran; 3 Non-Communicable Diseases Research Center, Sabzevar University of Medical Sciences, Sabzevar, Iran; 4 International UNESCO center for Health-Related Basic Sciences and Human Nutrition, Mashhad University of Medical Sciences, Mashhad, Iran; 5 Brighton & Sussex Medical School, Division of Medical Education, Falmer, Brighton, Sussex, BN1 9PH UK

**Keywords:** Depression, Quality of life, Lifelines diet score, Diet, Adolescents

## Abstract

**Background:**

It has been proposed that a greater degree of adherence to a healthy dietary pattern is associated with a lower risk of depression and a poor quality of life (QoL). The Lifelines diet score (LLDS) is a new, evidence-base scoring system to define the quality of diet. We designed a cross-sectional study to investigate the association between LLDS with depression and QoL in Iranian adolescent girls.

**Methods:**

A total of 733 female adolescents were recruited from Mashhad and Sabzevar cities, Iran. Depression and QoL were assessed utilizing the Beck Depression Inventory (BDI) and SF-12v2 questionnaires, respectively. The LLDS was defined by dividing intakes of 12 food groups with negative or positive health effects into quintiles ranging 12 to 60 points. To explore the association between LLDS with QoL and depression, logistic regression was used in crude and adjusted models.

**Results:**

The prevalence of depression and poor QoL was 24% and 49%, respectively. After adjusting for confounding factors, adolescent girls in the highest quartile of LLDS compared with the participants in the lowest quartile had a 42% lower probability of reporting depressive symptoms (OR: 0.58; 95% CI: 0.35–0.97, *P* = 0.03). In addition, the participants in the highest quartile of LLDS had lower odds of poor QoL compared with the subjects in the lowest quartile (OR: 0.65; 95% CI: 0.42–0.92, *P* = 0.04).

**Conclusions:**

There is an inverse relationship between LLDS with risk of depression and poor QoL. Prospective and interventional investigations are needed to reach a clear vision.

## Introduction

Depression is a common mental disorder with two main symptoms: depressed mood and anhedonia [1]. The worldwide prevalence of depression is estimated to be between 11.1 and 14.6% [2], with greater prevalence among adolescents and females [3]. Its prevalence among Iranian children and adolescents is estimated to be 43.5% [3]. The increasing prevalence of depression and its consequences such as suicide ideation and suicide attempts have led to elevated socioeconomic costs in both developed and developing countries [4–7]. Oxidative stress and inflammation are associated with psychological disorders [7, 8]. Depression is associated with an increased risk of obesity, type 2 diabetes mellitus (T2DM), cardiovascular disease (CVD), and infertility [9–11]. In addition, depression can lead to disrupted academic activities and motivation of students [12, 13]. The level of Quality of life (QoL), which is defined as “the perception of individuals about their position in life in the context of the culture and value systems which is related to their goals, expectations, standards and concerns” [14], decreases in patients of depression [15, 16].

Diet is a modifiable lifestyle factor that has been recognized as having an impact on mental wellbeing [17–19]. There is a relationship between a greater adherence to the western-style dietary patterns containing high amounts of red and processed meats, full-fat dairy products, saturated fatty acids (SFAs) and refined sugars with lower risk of mental disorders in adolescents [20, 21]. On the other hand, healthy dietary patterns rich in vegetables, fruits, sea foods, whole grains, legumes and nuts attenuate the risk of depression [22, 23].

Some studies reported the association between food groups and depression and quality of life. Results of the meta analysis study reported that fruit and vegetable consumption inversely associated with the risk of depression [24]. Another study has shown that vegetable and fruit intake are positively associated with health-related quality of life [25] Recently, the lifelines diet score (LLDS) was developed by Vinke et al. [26] to estimate the diet quality. This score is a food group-based diet quality assessment tool that reflects the latest international evidence on diet-disease associations. It has a high capacity to discriminate between people with widely different intakes. Moreover, it is flexible and widely applicable, as it uses a population-based quintile approach [26]. Other nutritional scores, such as the Healthy Eating Index and the Mediterranean Diet Score (MDS), are not entirely food-based and do not reflect the current scientific evidence [26]. Moreover, the MDS does not account for sugar-sweetened beverages, which have well-established detrimental effects on obesity and diabetes risk [27].

To the best of our knowledge, there is no study investigating the association between LLDS with depression and QoL. Accordingly, the present cross-sectional study was designed to determine the relationship between LLDS with depression and QoL in adolescent girls. We hypothesized that a higher adherence to the LLDS, demonstrating higher adherence to a healthy dietary pattern, may be associated with lower risk of depression and poor QoL.

## Methods and materials

### Study population

This cross-sectional study was performed in January 2015 in the cities of Mashhad and Sabzevar, Iran. The population of this study consisted of 733 female students aged 12–18 years recruited using a random cluster sampling method from different schools in these cities. Exclusion criteria included individuals with autoimmune disease, cancer, metabolic bone disease, hepatic or renal failure, cardiovascular disorders, malabsorption or thyroid, parathyroid, adrenal diseases, and anorexia nervosa or bulimia. Additionally, participants were excluded if they were taking any medication related to anti-inflammatory, anti-depressant, anti-diabetic, or anti-obesity drugs, vitamin D or calcium supplement use and hormone therapy within the previous 6 months [20]. The Ethics Committee of Mashhad University of Medical Sciences, Mashhad, Iran, approved the study and all participants and their parents provided informed written consent.

### Calculation of LLDS

Dietary intakes were evaluated using a validated and reliable food frequency questionnaire (FFQ) containing data on 147 food items [23]. This FFQ has nine multiple-choice frequency response categories that varies from “never or < 1/mo” to “≥12/d. FFQs were completed during face-to-face interviews and reported dietary intakes were converted to grams and entered to the Nutritionist IV software. To calculate daily nutrient intake values for each participant, the US Department of Agriculture’s (USDA) national nutrient databank was used [24]. The LLDS was computed by considering 12 food groups which had positive or negative health effects [26]. Vegetables, fruits, whole grain products, legumes and nuts, fish, oils and soft margarines, unsweetened dairy, coffee, and tea were classified as nine positive food groups and red and processed meat, butter and hard margarines, and sugar-sweetened beverages were defined as three negative food groups. The intake of food groups were adjusted by 1000 kcal energy intake and then divided in to quintiles from 1 to 5. The individuals in highest quintile of each positive food group’s intake received 5 points, while one point was specified to lowest quintile. For food groups with negative health effects, point 1 representing the maximum and point 5 representing the minimum dietary intakes. The sum of points for intakes of 12 food groups consisted LLDS ranging from 12 to 60 points.

### Assessment of depression

A Persian version of Beck Depression Inventory (BDI) was used to determine depression status in the current study. BDI is a self-administered questionnaire including 21 items with various options. The total score for the BDI ranges between 0 and 63 points. If the BDI score was < 13, the person was considered as not depressed, and if the subject’s score was > 13, they were categorized as depressed. The validity and reliability of BDI were assessed in previous studies (Cronbach’s α = 0.87 and acceptable test-retest reliability (*r*) = 0.74) [28].

### Quality of life assessment

To assess health-related quality of life, the SF-12v2 questionnaire was used. This questionnaire is a short form of the SF-36 questionnaire and improved version of SF-12v1 (7). The validity and reliability of this questionnaire was previously confirmed in Iran (8). The questionnaire has 12 items evaluating 8 domains of health including physical functioning, role limitations because of physical problems, bodily pain, general health, vitality, social functioning, role limitations because of emotional problems, and mental health. The range of quality of life scores are between 0 (the worst quality of life) to 100 (the best quality of life). The median of the quality of life score is 43. The subjects were considered as subjects with poor quality of life, if their scores were lower than 43.

### Covariate measurements

Weight and height were measured using standard protocols and the mean of two measurements was reported. Body mass index was calculated as weight (kg) divided by square of the height (m^2^). Mid-distance between the iliac crest and lowest rib was considered to measure waist circumference (WC). Hip circumference is measured at the largest circumference around the buttocks. Waist–hip ratio **(**WHR) is calculated as waist measurement divided by hip measurement. General Information including age, parental death or divorce was collected by face-to-face interview using a standard questionnaire. Physical activity was assessed through a validated modifiable activity questionnaire [22] and provided as metabolic equivalents (Mets) in hours per week.

### Statistical analysis

The normality of data was assessed using the Kolmogorov–Smirnov test. The LLDS was categorized into quartiles. To compare continuous and categorical variables across quartiles of LLDS, One-way Anova and Chi-square test were used, respectively. Logistic regression was applied to evaluate the relationship between LLDS with poor quality of life and depression prevalence in crude and adjusted models. Model I was adjusted for age and energy intake. Further adjustment was for BMI percentile. Physical activity, parent’s death and parent’s divorce were additionally adjusted in the model III. *P*-values < 0.05 were considered statistically significant. To analyze the data, statistical Package for Social Sciences (SPSS) (version 23.0, SPSS Inc., Chicago, Illinois, USA) was used.

## Results

### General characteristics study participants

The mean age of the participants was 14.5 years. The prevalence of depression and poor QoL was 24% and 49%, respectively. General characteristics and anthropometric variables of the participants across quartiles of LLDS are presented in Table 1. Age, percentile of BMI, WC, WHR, physical activity, parent’s death and parent’s divorce were not different among quartiles of LLDS. General characteristics and anthropometric variables of the participants across categories of depression and QoL score are presented in Table 2. The depressed subjects had more parents who divorced than those not depressed subjects. General characteristics and anthropometric variables were not different among participants with well quality of life and poor quality of life.

**Table 1:** General characteristics of study participants by quartile of LLDS

	Q1	Q2	Q3	Q4	*P* value*
Age(year)	14.36 ± 1.51	14.46 ± 1.49	14.57 ± 1.534	14.64 ± 1.58	0.324
Percentile BMI (kg/m2 )	44.10 ± 29.25	46.47 ± 27.74	49.21 ± 28.91566	51.33 ± 29.28	0.079
Waist circumference (cm)	69.20 ± 8.72	70.38 ± 8.17	70.97 ± 9.005	71.46 ± 10.16	0.106
Waist-to-hip ratio	0.76 ± 0.05	0.76 ± 0.05	0.76 ± 0.05877	0.77 ± 0.07	0.526
Physical activity (MET h/week)	45.21 ± 3.50	45.15 ± 3.58	45.49 ± 2.87713	45.61 ± 3.78	0.517
Parent’s death, *n* (%)	5 (2.8)	12 (7.6)	6 (3.2)	6 (3.1)	0.08
Parents’ divorce, *n* (%)	12 (6.8)	8 (5.2)	9 (4.8)	6 (3.1)	0.42
Quality of life score	41.70 ± 8.01	41.21 ± 7.85	41.88 ± 8.59058	43.32 ± 7.33	0.069
Depression score	11.94 ± 9.48	12.09 ± 9.48	10.96 ± 9.82	8.88 ± 7.99	< 0.001

BMI: Body mass index; LLDS: Life line diet score

Values are means ± SD and number (percent) for quantitative and qualitative variables, respectively.

*Obtained from one way Anova for continuous variables and Chi-squared test for categorical variables

**Table 2:** General characteristics of study participants by categories of depression and Quality of life score

	Not depressed	Depressed	*P* value*	Well quality of life	Poor quality of life	*P* value*	
Age(year)	14.51 ± 1.52	14.50 ± 1.56	0.917	14.45 ± 1.56	14.58 ± 1.51	0.266	
Percentile BMI (kg/m2 )	48.34 ± 29.31	46.29 ± 27.70	0.407	49.46 ± 29.79	46.01 ± 27.98	0.108	
Waist circumference (cm)	70.57 ± 8.88	70.29 ± 9.73	0.724	70.74 ± 8.88	70.23 ± 9.35	0.452	
Waist-to-hip ratio	0.76 ± 0.06	0.76 ± 0.06	0.493	0.76 ± 0.05	0.76 ± 0.06	0.815	
Physical activity (MET h/week)	45.45 ± 3.19	45.49 ± 3.95	0.856	45.62 ± 3.49	45.15 ± 3.40	0.065	
Parent’s death, *n* (%)	20 (3.70)	9 (5.10)	0.402	14 (3.00)	23 (5.200)	0.090	
Parents’ divorce, *n* (%)	17 (3.20)	18 (10.20)	< 0.001	17 (3.70)	27 (6.20)	0.085	
Quality of life score	44.10 ± 7.55	35.87 ± 5.75	< 0.001	48.45 ± 3.84	35.52 ± 5.25	< 0.001	
Depression score	6.54 ± 4.77	24.19 ± 6.57	< 0.001	6.29 ± 5.64	15.53 ± 9.86	< 0.001	

BMI: Body mass index

Values are means ± SD and number (percent) for quantitative and qualitative variables, respectively.

*Obtained from independent t-test for continuous variables and Chi-squared test for categorical variables

### Dietary intake study participants

Energy and dietary intakes of study participants by quartile of LLDS are shown in Table 3. Vitamin C (*P* < 0.01) and calcium (*P* = 0.01) intake were significantly higher among participants in the highest quartile of LLDS (per 1000 kcal) compared to the lowest quartile. The participants in the highest quartile of LLDS had higher intake of vegetables (*p* < 0.001), whole grain products (*P* = 0.04), fish (*P* < 0.01), oils and soft margarines (*P* = 0.02), tea (*P* < 0.01), coffee (*P* < 0.01) and unsweetened dairy (*P* = 0.04) compared to participants in the lowest quartile. Intake of cholesterol, saturated fatty acid, monounsaturated fatty acid, polyunsaturated fatty acid, iron, vitamin A, B6, B12 and folate were not significantly different between quartile of LLDS (per 1000 kcal). Food groups intake of study participants by across categories of depression and QoL score were presented in Table 4.

**Table 3:** Energy and dietary intakes of study participants by quartile of LLDS

	Q1	Q2	Q3	Q4	*P*-value*
Vegetables (gr)	191.47 ± 166.11	195.78 ± 153.39	210.32 ± 186.91	258.41 ± 176.96	< 0.001
Fruits (gr)	221.77 ± 255.90	215.94 ± 202.97	224.33 ± 204.41	269.56 ± 212.34	0.06
Whole grain products (gr)	55.06 ± 107.62	37.36 ± 66.38	62.56 ± 114.88	66.63 ± 105.54	0.04
Legumes and nuts (gr)	85.01 ± 66.87	84.07 ± 58.10	81.85 ± 66.28	98.37 ± 78.70	0.07
Fish (gr)	3.25 ± 5.21	2.72 ± 4.47	6.04 ± 16.45	5.29 ± 6.87	< 0.01
Oils and soft margarines (gr)	4.83 ± 6.20	6.42 ± 9.36	5.58 ± 6.69	7.32 ± 9.30	0.02
Unsweetened dairy (gr)	303.17 ± 239.76	355.96 ± 296.69	350.44 ± 248.45	383.87 ± 309.60	0.04
Coffee (gr)	2.39 ± 8.14	8.75 ± 24.81	11.03 ± 34.10	10.29 ± 21.52	< 0.01
Tea(gr)	302.75 ± 308.97	369.56368.96	412.31 ± 364.09	415.20 ± 334.51	< 0.01
Red and processed meat (gr)	21.81 ± 19.20	21.06 ± 22.27	20.17 ± 17.03497	18.50 ± 19.59	0.38
Butter and hard margarines (gr)	27.08 ± 30.40	29.17 ± 31.28	26.78 ± 26.73	26.66 ± 27.16	0.83
Sweetened beverages (gr)	124.01 ± 158.83	116.14 ± 159.10	104.13 ± 121.92	89.16 ± 119.82	0.08
Energy intake (Kcal)	1770.48 ± 819.72	2700.37 ± 830.22	2960.94 ± 829.97	2691.51 ± 846.69	0.76
Carbohydrate (% energy)	55.65 ± 7.84	53.94 ± 7.61	55.05 ± 6.86	54.58 ± 6.89	0.16
Protein (% energy)	13.54 ± 2.23	13.43 ± 2.29	13.68 ± 2.06	13.58 ± 2.31	0.31
Fat (% energy)	33.12 ± 7.97	34.91 ± 8.29	33.47 ± 7.18	33.96 ± 7.36	0.15
Cholesterol (mg/1000 Kcal)	84.46 ± 41.51	92.05 ± 59.94	89.62 ± 41.06	91.40 ± 38.89	0.38
Saturated fatty acid (gr/1000 Kcal)	10.77 ± 3.22	11.52 ± 3.55	11.18 ± 3.25	11.45 ± 3.38	0.14
Monounsaturated fatty acid (gr/1000 Kcal)	11.84 ± 3.37	12.73 ± 3.93	11.93 ± 3.19	12.09 ± 3.35	0.08
Polyunsaturated fatty acid (gr/1000 Kcal)	8.12 ± 3.04	8.89 ± 3.70	8.29 ± 2.71	8.28 ± 3.19	0.12
Calcium (mg/1000 Kcal)	399.92 ± 129.64	426.51 ± 147.24	432.53 ± 127.60	444.10 ± 132.81	0.01
Iron (mg/1000 Kcal)	7.54 ± 1.50	7.33 ± 1.52	7.36 ± 1.45	7.29 ± 1.40	0.38
Vitamin A (mcg/1000 Kcal)	187.42 ± 108.81	248.89 ± 468.07	216.36 ± 100.85	245.65 ± 119.17	0.05
Vitamin C (mg/1000 Kcal)	32.18 ± 18.64	33.77 ± 18.52	35.84 ± 22.27	40.11 ± 21.28	< 0.01
Folate (mcg/1000 Kcal)	224.83 ± 45.12	226.89 ± 53.92	230.78 ± 47.33	230.17 ± 50.68	0.61
Vitamin B6 (mg/1000 Kcal)	0.71 ± 0.13	0.69 ± 0.11	0.71 ± 0.12	0.71 ± 0.13	0.15
Vitamin B12 (mcg/1000 Kcal)	1.43 ± 0.76	1.87 ± 4.75	1.52 ± 0.69	1.56 ± 0.70	0.29

LLDS: Life line diet score

Values are means ± SDs

*Obtained from One way Anova

**Table 4:** Food groups intake of study participants by categories score of depression and quality of life

Categories of depression score	Not depressed	Depressed	*P*-value*
Vegetables (gr)	220.96 ± 177.48	199.11 ± 162.51	0.142
Fruits (gr)	240.71 ± 214.53	214.05 ± 237.08	0.157
Whole grain products (gr)	58.66 ± 103.04	49.25 ± 98.76	0.281
Legumes and nuts (gr)	88.99 ± 70.56	83.27 ± 62.14	0.330
Fish (gr)	4.87 ± 10.92	3.09 ± 5.49	0.035
Oils and soft margarines (gr)	6.07 ± 7.75	5.87 ± 8.84	0.770
Unsweetened dairy (gr)	342.94 ± 273.56	329.88 ± 267.62	0.575
Coffee (gr)	7.51 ± 21.51	9.82 ± 30.75	0.264
Tea(gr)	382.10 ± 352.25	353.92 ± 328.03	0.342
Red and processed meat (gr)	19.48 ± 18.92	18.70 ± 17.09	0.622
Butter and hard margarines (gr)	27.54 ± 28.76	26.73 ± 28.83	0.729
Sweetened beverages (gr)	58.47 ± 77.43	61.44 ± 76.17	0.653
Categories of quality of life score	Well quality of life	Poor quality of life	
Vegetables (gr)	223.56 ± 184.81	209.09 ± 163.53	0.264
Fruits (gr)	255.50 ± 217.29	214.91 ± 223.35	0.013
Whole grain products (gr)	58.75 ± 96.53	52.47 ± 100.78	0.391
Legumes and nuts (gr)	87.85 ± 69.04	87.49 ± 68.66	0.943
Fish (gr)	4.35 ± 6.63	4.54 ± 12.39	0.797
Oils and soft margarines (gr)	6.51 ± 8.34	5.62 ± 7.73	0.137
Unsweetened dairy (gr)	358.96 ± 292.96	319.79 ± 249.77	0.053
Coffee (gr)	6.58 ± 18.94	9.71 ± 28.55	0.082
Tea(gr)	370.21 ± 334.97	383.77 ± 359.61	0.599
Red and processed meat (gr)	18.93 ± 16.65	19.71 ± 20.30	0.570
Butter and hard margarines (gr)	27.92 ± 28.91	26.93 ± 28.81	0.646
Sweetened beverages (gr)	53.90 ± 65.12	64.46 ± 87.64	0.066

Values are means ± SDs

*Obtained from One way Anova

The association between LLDS with depression and poor QoL.

Multi-variable adjusted odds ratios (ORs) for depression and poor QoL categories across quartiles of LLDS are represented in Table 5. Adolescent girls in the highest quartile of LLDS compared with the participants in the lowest quartile had a 41% lower probability of having depressive symptoms (OR: 0.59; 95% CI: 0.36–0.96, *P* = 0.03). This association remained significant after adjustment for age, energy intake, BMI percentile, physical activity, parent’s death and parent’s divorce (OR: 0.58; 95% CI: 0.35–0.97, *P* = 0.03). The participants in the highest quartile of LLDS had lower odds of poor QoL compared with the subjects in the lowest quartile (OR: 0.62; 95% CI: 0.41–0.94, *P* = 0.02) in crude model. This association remained significant after adjustment for age, energy intake, BMI percentile, physical activity, parent’s death and parent’s divorce (OR: 0.65; 95% CI: 0.42–0.92, *P* = 0.04).

**Table 5:** Multivariable-adjusted odds ratio of the associations between LLDS with poor quality of life and depression

	Q1	Q2	Q3	Q4	*P* value¹	*P* trend
Poor quality of life						
Crude	1.00	0.98(0.64_1.49)	0.80(0.53–1.21)	0.62(0.41–0.94)	0.025	0.016
Model1	1.00	0.98(0.64_1.50)	0.80(0.53–1.20)	0.61(0.40–0.92)	0.020	0.013
Model2	1.00	0.99(0.65_1.52)	0.82(0.54–1.23)	0.63(0.41–0.95)	0.028	0.019
Model3	1.00	1.00(0.64_1.54)	0.83(0.55–1.27)	0.65(0.42–0.99)	0.048	0.036
Depression						
Crude	1.00	1.13(0.71_1.79)	0.86(0.54–1.37)	0.59(0.36–0.96)	0.034	0.022
Model1	1.00	1.13(0.71_1.81)	0.85(0.53–1.35)	0.57(0.35–0.93)	0.026	0.016
Model2	1.00	1.14(0.72_1.81)	0.86(0.54–1.37)	0.58(0.35–0.94)	0.030	0.020
Model3	1.00	0.95(0.58_1.54)	0.81 (0.50–1.30)	0.58(0.35–0.97)	0.036	0.033

LLDS: Life line diet score

¹Last quartile compared to first quartile

Model 1: Adjusted for age and energy intake

Model 2: Additionally, adjusted for BMI percentile

Model 3: Additionally, adjusted for physical activity, parent’s death and parent’s divorce

## Discussion

The findings of our cross-sectional study showed that higher LLDS is associated with lower prevalence of depression in adolescent girls. An inverse correlation was found between LLDS and poor QoL. Depression can adversely affect academic attainment and acquisition of effective social skills, decrease motivation, and increase suicide ideation as well as suicide attempts in adolescents [6, 7, 12, 13]. Adherence to the healthy dietary patterns can decrease the risk of depression and poor QoL [29, 30]. The LLDS is a new, flexible and evidence-base score used to estimate adherence to a healthy diet [26]. A higher LLDS indicates higher consumption of positive food groups (vegetables, fruits, whole grain products, legumes and nuts, fish, oils and soft margarines, unsweetened dairy, coffee, and tea) and lower consumption of negative food groups (red and processed meat, butter and hard margarines, and sugars sweetened beverages), and vice versa [26]. To the best of our knowledge, this is the first study that examined the association of LLDS with depression and QoL in adolescent girls. We found that higher level of LLDS is associated with lower risk of depression and poor QoL.

Subjects with higher LLDS consumed higher amounts of plant foods. In general, plant foods and their biological compounds regulate dopaminergic pathways of brain, increase the levels of serotonin and dopamine in the hippocampus and prefrontal cortex, decrease the activity of monoamine oxidase (MAO) and subsequent improve depression [31–34]. In line with our findings, a cross-sectional study demonstrated that greater adherence to dietary approaches to stop hypertension (DASH), which is a healthy plant-based dietary pattern, is associated with lower odds of depression [30]. Likewise, it has been reported that risk of depression is lower in subjects with higher adherence to MDS (a plant-based diet) [22].

Oxidative stress and inflammation are two factors linked to the development of depression [8, 35]. Biological components of plant foods such as phytochemicals, which have antioxidant and anti-inflammatory properties, can modulate the production of fatty acids, improve mitochondrial dysfunction, increase the levels of antioxidant factors, decrease production of free radicals and pro-inflammatory cytokines [36–39]. The cross-sectional study of Holt et al. [40] demonstrated an inverse relationship between consumption of plant foods with levels of oxidative stress and inflammation. In addition, we demonstrated that participants in higher quartile of LLDS had higher intake of Vitamin C. Vitamin C through mechanisms such as inhibiting the activity of hydroxyl radical, tocopheroxyl radical, peroxyl radical, thiol radical and alkoxyl radicals by providing an electron for these damaging oxidizing radicals, decreasing macrophages-induced LDL oxidation, regulating nitric oxide-cyclic GMP (cGMP) pathways, inhibiting potassium channels, activating γ-aminobutyric acid (GABA) type A receptors and decreasing GABA type B receptors activation, decreasing levels of tumor necrosis alpha (TNF-α) and interleukins in the hippocampus and decreasing the activity of nuclear factor κ-B (NF-κB) and mitogen-activated protein kinase (MAPK) pathways exert its antidepressant-like effect [41–47]. A study confirmed that sub-optimal vitamin C status is associated with increased symptoms of depression [48].

As mentioned, we found an inverse association between LLDS and QoL. There is no other study investigating this relationship. However, some studies have demonstrated a direct association between adherences to the healthy diets with QoL. As shown, two cross-sectional studies have reported that adherence to the Mediterranean diet is directly associated with QoL [49, 50]. Moreover, diets with higher quality containing higher amounts of whole grains, fruits and vegetables as well as lower amounts refined foods and fast food are associated with better QoL [51–53]. Recent systematic review of Govindaraju et al. [54] revealed a direct association between adherence to the dietary patterns rich in whole grains, fruits and vegetables with QoL.

There are some strength points for our study. This study was conducted in a large sample and is one of the first studies to examine the relationship between LLDS and depression or QoL in adolescent girls. High quality of data collection by validated questionnaires was first strength point in the current study. In addition, to avoid misleading conclusions in analysis and interpretation of data, we conducted rigorous statistical analyses, including several adjustment models for confounding factors to depression and QoL. Nevertheless, our findings require to be interpreted by considering some potential limitations. Cross-sectional design should be considered as the major limitation of the present study; because, we do not confirm a causal relationship. FFQs are prone to measurement error and misclassification. Also, the current study was performed only on girls and not boys that this might be a limitation. Finally, like other observational studies, several unmeasured confounders were in this study, which we are unable to control them.

## Conclusions

In summary, findings of the current study suggest that a greater adherence to LLDS was associated with lower odds of depression and poor QoL in adolescent girls. Children and their parents should be informed which higher dietary intakes of antioxidant and fiber sources and lower consumption of saturated fatty acids and sugar promote mental health. In addition, adolescent-centered nutritional interventions should be designed as a school snack with a greater emphasis on fruits, vegetables, nuts, legumes, whole grain and dairy products. These findings are needed to confirm with prospective studies and trials to clarify the causal association and determine the dose-response curve between diet quality and risk of depression or poor QoL.

## Data Availability

The data and materials of the current study is available from the corresponding author on reasonable request.

## Abbreviations

ALT: Alanine transaminase
AST: Aspartate transaminase
BDI: Beck depression inventory
BMI: Body mass index
cGMP: Cyclic GMP
FFQ: Food frequency questionnaire
FPG: Fasting plasma glucose
GABA: γ-aminobutyric acid
GGT: Gamma-glutamyl transpeptidase
HDL-c: High density lipoprotein-cholesterol
LDL-c: Low density lipoprotein-cholesterol
MAO: Monoamine oxidase
MAPK: Mitogen-activated protein kinase
MAQ: Modifiable activity questionnaire
NF-κB: Nuclear factor κ-B
OR: Odds ratio
QoL: Quality of life
SD: Standard deviation
SPSS: Statistical package for social science
TC: Total cholesterol
TG: Triglyceride
WC: Waist circumference
